# The Role of Selected Myokines in the Development of Cardiovascular Diseases, and Their Involvement in Developing Heart Failure in Rheumatoid Arthritis Patients

**DOI:** 10.3390/ijms26178194

**Published:** 2025-08-23

**Authors:** Jakub Kuna, Grzegorz Chmielewski, Łukasz Jaśkiewicz, Michalina Knapik, Magdalena Krajewska-Włodarczyk

**Affiliations:** 1 Department of Rheumatology, School of Medicine, Collegium Medicum, University of Warmia and Mazury in Olsztyn, Al. Wojska Polskiego 30, 10-229 Olsztyn, Poland; kuna.jakub@wp.pl (J.K.); gchmielewski.gc@gmail.com (G.C.); michalina24knapik@gmail.com (M.K.); 2 Department of Human Physiology and Pathophysiology, School of Medicine, Collegium Medicum, University of Warmia and Mazury in Olsztyn, ul. Michała Oczapowskiego 2, 10-719 Olsztyn, Poland; lukasz.jaskiewicz@uwm.edu.pl

**Keywords:** myokines, myonectin, irisin, musclin, follistatin-like1 (FSTL1), dermcidin, apelin, myostatin, hypertension, heart failure, coronary heart disease, rheumatoid arthritis

## Abstract

Cardiovascular diseases, which are among the most common diseases of the population and among the leading causes of death, are a constant topic of many research centers. A deeper understanding of their pathogenesis may contribute to the development of innovative diagnostic and therapeutic techniques. Recently, the role of myokines—a group of cytokines secreted mainly by muscle cells—has been increasingly emphasized in the development of these diseases. Both their excess and deficiency can cause undesirable effects that are involved in the pathomechanism of these diseases. In this review, we focus on the latest studies on the role of myonectin, irisin, musclin, follistatin-like1 (FSTL1), dermcidin, apelin, and myostatin in the pathogenesis of coronary artery disease, heart attack, heart failure, and hypertension. In particular, we look at myostatin and irisin in the context of the development of heart failure and decreased levels of apelin with higher cardiovascular risk in a group of patients with rheumatoid arthritis.

## 1. Introduction

Cardiac diseases—mainly hypertension, heart failure, coronary heart disease (especially aggravated by acute coronary syndromes)—are widespread illnesses, the latter being the most frequent cause of death in the adult population [1,2]. Recent years abound in reports helpful in understanding the pathophysiology of these diseases; one of the more promising avenues is the analysis of the physiological and pathological influence of myokines, both on the heart muscle cells and blood vessels. Myokines, first referred to with that name in 2003 by Pedersen et al. [3,4], are a group of cytokines produced predominantly by skeletal muscles (among them are cardiokines, produced by the heart muscle cells [5]). The focus of Pedersen’s research was on interleukin-6 (IL-6) as a possible mediator between the exercising muscle and distant organs. The first myokine identified as originating primarily from skeletal muscle, rather than other tissues, was myostatin. Since then, hundreds of these peptides have been identified, yet a biological role has been found for only a small percentage of them. Myokines exhibit auto-, para-, and endocrine properties, and their receptors have been found in skeletal muscles, bones, liver, pancreas, brain, adipose tissue, as well as within the heart. They perform numerous functions, including, but not limited to regulation of tissue metabolism, promotion or inhibition of tissue growth (mainly muscle and bone), hormonal regulation (insulin, cortisol), modulation of the immune response, or anti-cancer activity. Lately, another term has been proposed, that would encompass the cytokines released by multiple organs during the exercise: exerkines. Myokines are a subset of this larger family [3].

Studies in recent years have distinguished numerous myokines exhibiting direct or indirect influence on the heart. This paper focuses on myonectin, irisin, musclin, follistatin-like1 (FSTL1), dermcidin, apelin, and myostatin. Their roles in particular heart pathologies are shown in Table 1.

Rheumatoid arthritis (RA) constitutes an independent predictor of an increased risk of cardiovascular incidents, developing heart failure (HF), and skeletal muscle dysfunction. Two of the myokines, myostatin and irisin, are involved in negative outcomes both in RA and HF, and decreased levels of apelin predicted higher cardiovascular risk in this group of patients. It is known that myostatin, a myokine of the TGF-β infraorder, negatively regulates muscle growth and fosters diversification of osteoclasts, which may amplify the destruction of joints caused by RA. A high concentration of myostatin is connected with an increased progression of radiographic changes in patients with RA [6]. It has been observed that another myokine, irisin, may modulate the immune response; it has been demonstrated to decrease the markers of inflammation in experimental models of arthritis, which suggests potential therapeutic benefits [7]. A decreased level of irisin has been observed in patients with RA and correlated with disease activity, its duration, and the duration of morning stiffness. Poor sleep quality further lowered its level [8].

Several studies did show a decrease of apelin levels in RA patients. Although apelin was connected with promotion of metalloproteinases production when administered in vitro to chondrocytes (mainly MMP-1, MMP-3, and MMP-9) and showed positive correlation with MMP-2 in patients with RA, therefore facilitating harmful effects on joint structure, its reduced levels in RA were predictors of development of atherosclerosis and plaque instability, leading to higher cardiovascular risk in this group of patients [9].

There is numerous conflicting evidence of the predictive value of several myokines, mainly in heart failure with reduced ejection fraction (HFrEF) [10]. A higher concentration of irisin in the serum has been observed in patients who died during the one year observation period following an acute heart failure episode (higher concentrations of irisin are frequently observed in patients with volumetric overload and active inflammatory processes in the myocardium) [11]. Additionally, there is a substantial positive correlation between increased irisin levels and clinical results in patients with diagnosed cardiovascular disease following a myocardial infarction, independent from the presence of heart failure [12]. An increased concentration of irisin in the serum was a strong predictive biomarker of both total mortality and mortality caused by cardiovascular diseases within one year period for patients with acute and chronic HF [13]. Myostatin is an independent prognostic factor for mortality in patients with HF and those requiring re-hospitalization due to progressing HF [14].

To date, precise models of molecular trails corresponding to the link between myokines and the development of particular HF stages have not been presented. However, in the future myokines may become a new target of clinical therapy in HF patients of different phenotypes, especially in patients with HF and concurrent metabolic diseases.

## 2. Role of Selected Myokines in Cardiovascular Diseases

### 2.1. Dermcidin

Dermcidin (DCD) was first discovered in 2001, initially as a protein produced by perspiratory glands; it exhibited antimicrobial properties [15]. Its expression in other organs was detected in the following years [16,17,18]. DCD’s role in the pathophysiology of some types of solid tumors was also documented: it was observed to induce cell multiplication and migration, as well as increase survivability in cases of hypoxia [19,20,21].

In persons diagnosed with coronary artery disease, chronic peripheral vascular disease is a significant independent factor for poor prognosis, and the risk of cardiovascular incidents increases three- to six-fold [22,23,24]. Hitherto, suspicions that both these diseases are only a joint manifestation of generalized atherosclerosis have been undermined by recent studies [23,25].

In a study on mice with no indicators of atherosclerosis, which was published in 2015 [22], ischemia of the hind limbs was artificially induced, resulting in a significant increase in the concentration of myokines, including DCD. Its high levels correlated with an increase in the area of ischemia and a faulty reconstruction of the myocardium after an infarction. The study did not demonstrate a significant difference in the concentration of proinflammatory cytokines between the group with prior induced ischemia of the hind limbs and that without ischemia. DCD may thus independently sensitize cardiomyocytes to apoptosis, although it is unknown in what mechanism that may occur. In the case of healthy cardiomyocytes with no risk of damage, DCD did not induce apoptosis [22].

Another study showed elevated dermcidin levels in patients with acute coronary syndrome and acute myocardial infarction (they were significantly higher in the latter condition). The same study revealed dermcidin to be a potent NO synthesis inhibitor in the blood platelets and to bind to the platelet surface, facilitating their increased aggregation. This effect profoundly impeded the antiplatelet aggregation effects of aspirin, a drug commonly used during the initial stages of myocardial infarction. Moreover, higher dermcidin concentration in plasma may contribute to partial resistance of the platelets to aspirin. The authors mention insulin as a potential molecule that can reverse this effect and also suggest considering higher doses of aspirin during myocardial infarction to overcome this dermcidin effect [26].

**Figure 1:**
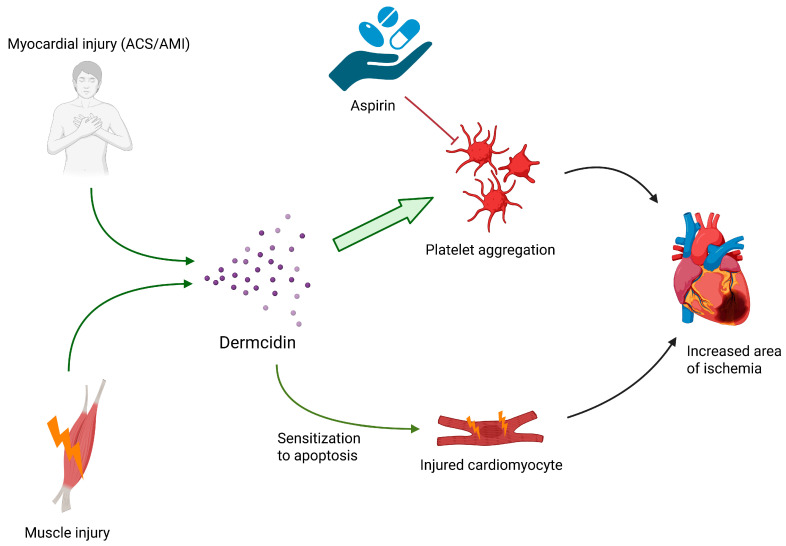
Dermcidin concentration is induced by myocardial injury and skeletal muscle injury. Dermcidin sensitizes cardiomyocytes to apoptosis and acts as a strong factor for platelet activation capable of overcoming aspirin effects; both actions increase the area of ischemia in myocardial infarction.

The summary of dermcidin’s aforementioned functions are displayed in Figure 1. 

### 2.2. Irisin

Irisin is a particle comprised of 112 amino acids, originating from the transmembrane protein of muscle cells. It is cleaved from N-terminal portion of fibronectin type III domain-containing protein 5 (FNDC5) that is a membrane-spanning protein. It is known that exercise and shivering induced by cold upregulate irisin in the circulation. Moreover it has been observed that synthesis of FNDC5 is diminished by fatty acids, hyperglycemia, and inflammatory molecules (such as IL-1 beta or TNF-alpha) [27]. Irisin plays a role in appetite regulation via brain-derived neurotrophic factor (BDNF), and interacts with adipose tissue, causing it to brown [28,29,30]. Overexpression of irisin caused a decrease of insulin resistance in animal models of diabetes [30]. Together with other myokines secreted by skeletal muscles during physical exertion, such as IL-6, -8, -15, fibroblast growth factor (FGF), and BDNF, irisin regulates the functioning of the cardiovascular system [31]. It has been demonstrated that cardiomyocytes also produce irisin in higher quantities than the skeletal muscle cells [32]; its high concentration was reported in cases of myocardial ischemia, volumetric overload, and inflammation [33]. The protein has been proved to play a significant role in the pathogenesis of myocardial infarction, heart failure, and arterial hypertension [28].

Lower concentrations of circulating irisin have been observed in patients with stable coronary disease and advanced atherosclerotic lesions [34], which has also been demonstrated through meta-analysis of patient observations between 2000 and 2017, where patients with coronary disease exhibited lower concentrations of irisin compared to healthy persons [35]. The concentration of irisin was significantly higher in patients with diabetes, without coronary disease, than in those with both diabetes and coronary disease [36]. Some studies report a positive correlation between an increased concentration of irisin and the risk of coronary disease, acute coronary incidents, and the development of heart failure [12,37].

Irisin plays an important role in maintaining mitochondrial homeostasis following a myocardial ischemic incident. There is also a correlation between irisin concentration and different repair phases following an acute coronary syndrome [12,38,39]. Irisin achieves the highest concentration approximately 8 h after STEMI, which then gradually decreases over three days after the infarction [12]. In early stages, oxygen-based free radicals (ROS) are released after reperfusion and the complement system is activated, which triggers the recruitment of leukocytes and macrophages, as well as the overexpression of inflammatory cytokines (such as TNF-a, IL-1B, IL-6, IL-18). Administering irisin at this stage improves the functioning of mitochondria and protects myocytes from damage caused by hypoxia and reperfusion injury [40]. Beside the positive effects of irisin on the myocardium during this early stage, it has also exhibited a protective effect on the endothelium [41]. Within 3–5 days after an infarction, the inflammatory response is significantly diminished, and repair processes begin—the formation of post-infarction scarring, myocyte reconstruction, and neovascularization. Immunomodulatory and anti-inflammatory processes occur until around the 14th day. In mouse models, exhausting irisin reserves at this point caused an increase in the concentration of proinflammatory cytokines (such as IL-6 or TNF-a) and a decrease of IL-10 [42]. In mice with induced irisin overexpression, increased mitochondrial activity led to a higher production of ROS—a useful process in the acute phase, but which was detrimental in the later phase, where in hypoxic conditions ROS induced the process of myocyte apoptosis [43]. In a study measuring irisin concentration in patients after various time intervals following STEMI, its increased concentration one month after an ischemic incident correlated with a heightened risk of acute heart failure episodes [12]. A persisting long-term high concentration of irisin in those patients may suggest that the inflammatory process was still ongoing, and the high irisin level was a secondary symptom of an existing inflammation [12].

Patients with heart failure exhibit abnormalities in the structure and functioning of mitochondria (lowered efficiency of energy functions), deterioration of fatty acid oxidation processes, and a handicap of peripheral oxygen diffusion mechanisms [44,45]. One important factor regulating the process of creating new morphologically correct mitochondria and significantly affecting the correct function of the heart is peroxisome proliferator-activated receptor gamma coactivator 1-alpha (PGC-1α), the concentration of which increases during physical exertion [46,47] and decreases during heart failure [48,49]. Irisin is a myokine dependent on the concentration of PGC-1α [30]. To date, there has been one study reporting a higher concentration of irisin in cases of heart failure with preserved systolic function (HFpEF) than in cases with reduced systolic function (HFrEF) [50]. There has been found a positive correlation of irisin concentration with type-B natriuretic peptide (BNP) and heart failure class according to NYHA [51]. An increased level of irisin may heighten the oxygen demand in cardiomyocytes and aggravate oxidation stress [52]. Chronic heart failure is often accompanied by cachexia, which in turn means that a low irisin concentration may be connected with sarcopenia, so its predictive value diminishes in this case (although it can serve as a marker of progressing decrease in muscle mass) [52]. Currently, there are no conclusive studies on the connection between irisin and heart failure, if only due to the multitude of variations and phases of heart failure and the acuteness of its course. Although irisin cannot be unambiguously treated as a biomarker of heart failure, its high concentration in patients with HF is a predictor of poor prospects, including sudden cardiac death within one year [11].

In the case of arterial hypertension, the mechanisms and influence of irisin are not yet clear. In persons exercising regularly, the average concentration of irisin is higher, and the resting systolic and diastolic blood pressure is lower [53]. Some studies have demonstrated a positive correlation between the irisin level and arterial blood pressure in persons without arterial hypertension [54]. A negative correlation between the irisin level and arterial blood pressure has been observed during pre-eclampsia [55]. In turn, patients taking antihypertensive drugs exhibited heightened irisin levels [56].

**Figure 2:**
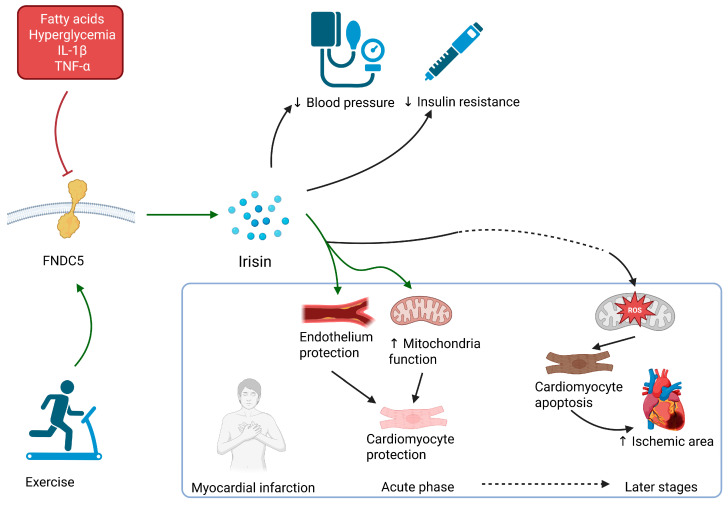
Irisin is cleaved from FNDC5 muscle membrane protein. Its expression is induced by exercise and hampered by fatty acids, hyperglycemia, and inflammatory molecules. It is known to lower blood pressure and overcome insulin resistance. During myocardial infarction, it acts protectively to cardiomyocytes by endothelium protection and facilitating of mitochondria function. When overexpressed in later stages of MI, it can promote myocyte apoptosis through overstimulation of mitochondrial functions. IL-1β: interleukin-1-beta; TNF-α: tumor necrosis factor alpha; ↑ increase; ↓ decrease.

The summary of the irisin’s functions are displayed in Figure 2.

### 2.3. Musclin

Known for approximately 20 years now, musclin is a peptide produced by muscles, but also present in osteoblasts [57,58,59]. It is coded by the *Ostn* gene sequence expressed in those cells. Musclin contains regions similar to natriuretic peptides, particularly type-C (NPR-C) [60].

A higher musclin expression has been detected in the arteries of mice with hypertension. This has been confirmed by experiments on mice with hypertension induced by deoxycorticosterone acetate and sodium chloride (DOCA-salt), as well as mice after multiple vasoconstrictions induced by phenylephrine. Similar effects have been observed in mice with hypertension caused by removing a kidney [57].

It is assumed that an overexpression of musclin may amplify the symptoms of hypertension through vasoconstriction. Such a process is dependent on the availability of calcium. The condition of the vascular endothelium (considered to be key in regulating vascular constriction) had no bearing on inducing the constrictions in the above experiments [57].

Similarly indirect clues hint at musclin playing a role in the pathogenesis of arterial hypertension owing to its similarity to NPR-C, whose known function is the removal of natriuretic peptides responsible for lowering arterial blood pressure [58,61].

It has been shown that musclin plays an important role in cardiomyocyte protection. In response to physical activity musclin induces biogenesis of cardiac mitochondria: after physical exercise elevated musclin levels facilitated atrial natriuretic peptide (ANP)/cGMP signaling in skeletal and heart muscle, and cGMP has been shown to upregulate PGC1 alpha-dependent mitochondrial generation in many tissues, including the heart. Moreover, this mitochondria-synthesis effect was shown after intravenous administering of musclin in mouse models [62].

Low skeletal muscle mass, such as seen in patients with chronic HF, further induces HF progression. In skeletal muscles of a mouse model of cardiac cachexia, a reduced expression of *Ostn* has been found. That same deficiency was found in skeletal muscle biopsies of patients with chronic HF. In a study conducted by M. Szaroszyk et al. [63]. published in 2022, different murine models with heart failure induced by chronic transverse aortic constriction have been used. This procedure facilitated cardiac hypertrophy, pulmonary congestion, reduced LVEF, and reduction in skeletal muscle mass (therefore reduced musclin expression). Overexpression of musclin by administering adeno-associated virus 6 (AAV6) improved left ventricular function and reduced muscle fibrosis. Authors postulate this effect may have been achieved due to improved cardiomyocyte contractility signalled by CNP/NPR-B and inhibition of fibroblast proliferation by activation of protein kinase G (PKG) [63].

In the light of the above studies, musclin may provide a potential therapeutic avenue, especially in patients with low skeletal muscle mass.

**Figure 3:**
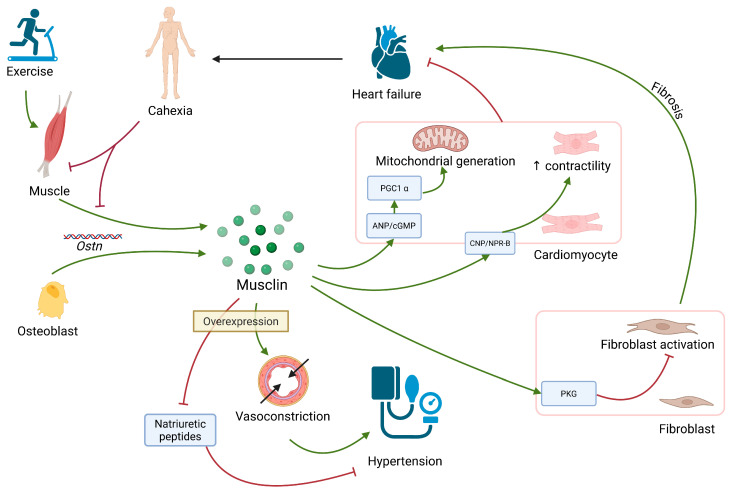
Musclin is expressed in muscle tissue and osteoblasts. Exercise stimulate its expression; in cachexia (often present in heart failure), lower expression of its coding gene, *Ostn*, is found. It inhibits fibroblasts activation through PKG; therefore, it suppresses fibrosis. In cardiomyocytes, it promotes contractility and stimulates mitochondrial generation. Those actions inhibit heart failure. When overexpressed musclin causes vasoconstriction and acts in opposition to natriuretic peptides, both of those actions promote hypertension. PKG: protein kinase G; ANP/cGMP: atrial natriuretic peptide/cyclical GMP; PGC1α: peroxisome proliferator-activated receptor gamma coactivator 1-alpha; CNP: C-type natriuretic peptide; ↑ increase.

Figure 3 contains the summary of musclin’s actions.

### 2.4. Myonectin

Myonectin plays a role in the heart remodeling process after an infarction. It is also known as C1q/TNF-related protein. Its expression increases during physical exertion. Unlike myokines such as FSTL-1, IL-6, or IL-15, whose increased levels can be observed during resistance training [64,65,66,67], myonectin levels increase during endurance training [68]. Beside the skeletal muscle, myonectin has also been located in hepatocytes, where it causes the suppression of autophagic processes [69]. In mice, it increases the uptake of fatty acids by adipocytes and hepatocytes, decreasing the level of circulating free fatty acids [68,69].

In tests performed on mice, a lowered myonectin concentration correlated with larger necrosis areas in cardiac infarction, more severe heart failure, and longer inflammation duration in the period of post-infarction reperfusion. In turn, myonectin overexpression measured in skeletal muscles correlated with a significantly smaller area of ischemia: in this experiment, a higher expression of myonectin was triggered through physical exertion. In mouse models with reduced myonectin expression not affected by physical effort, larger areas of post-infarction ischemia were observed [68].

It is postulated that the cardioprotective property of myonectin is induced through stimulation of kinase B protein (Akt)-dependent signaling in the heart. Akt inhibits the apoptosis of cardiomyocytes and lowers the post-inflammatory response of macrophages [70,71]. Myonectin increases the level of sphingosine-1-phosphate (S1P) in macrophages and cardiomyocytes. Blocking S1P interrupts the intracellular signal, which in normal conditions is responsible for diminishing the inflammatory effects of macrophages and the apoptosis of myocytes [68].

Another study proposed one more cardioprotective function of myonectin: in a mouse model with hind-limb ischemia, myonectin was shown to promote angiogenesis, especially in ischaemic conditions. By acting on the endothelial cells it facilitated formation of capillary-like structures, promoted their migration and proliferation. Those features were present in both in vitro and in vivo experiments [72].

**Figure 4:**
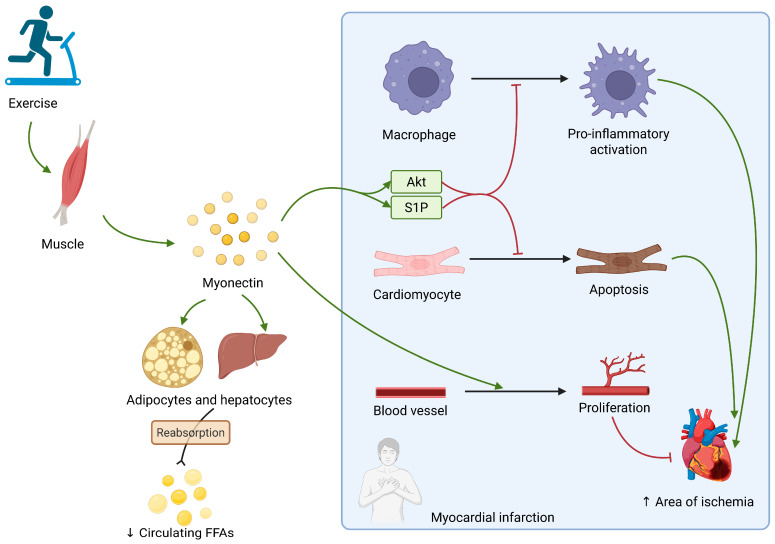
Myonectin, a muscle-derived myokine, acts on adipocytes and hepatocytes, promoting resorption of FFAs. During myocardial infarction it inhibits pro-inflammatory macrophage activation and cardiomyocyte apoptosis through Akt and S1P. It also promotes blood vessel proliferation. All of the above reduce the area of ischemia. FFAs: free fatty acids; Akt: kinase B protein; S1P: sphingosine-1-phosphate; ↑ increase; ↓ decrease.

The summary of myonectin’s aforementioned functions is displayed in Figure 4.

### 2.5. Apelin

Apelin, or the apelin receptor system, is one of the domains regulating arterial blood pressure and the functioning of the cardiovascular system. Its activation widens the blood vessels, triggers antiplatelet mechanisms, increases diuresis, and has a positive inotropic effect. Apelin, beyond being classified as a myokine, is also considered an adipokine [9,73,74,75,76,77]. Apelin receptors were found in skeletal muscles, cardiomyocytes, in the vascular endothelium, and also in the brain, kidney, lung, and spinal cord. Apelin receptors are expressed alongside angiotensin AT1 receptors and the apelin system acts in opposition to the renin–angiotensin system. It is worth mentioning that the apelin receptors are also activated by a second peptide, elabela, yet the signaling pathway of elabela and apelin receptor interaction has not been yet studied. Apelin knockout animals develop normally, but those lacking the apelin receptor show signs of cardiovascular defects, usually fatal in embryonic states. Elabela deficiency also leads to similar outcomes as in animals lacking the receptor [78,79,80].

A decreased concentration of apelin has been observed in patients with arterial hypertension aggravated by left ventricular hypertrophy, as well as those with ischemic heart disease [81,82]. In patients with chronic heart failure, administering pyr1-apelin-13 increased the cardiac index and decreased peripheral vascular resistance [76,83].

Another effect of a lowered apelin concentration, observed in mouse models, is the hypertrophy of the myocardium accompanied by decreased capillary density. The loss of apelin resulted in a heightened activity of angiotensin 2, which in turn increased myocardial fibrosis and caused a surge in the transcriptive activity of profibrotic mediators, leading to the deposition of collagen I and III [73].

In individuals with hypertension both apelin, elabela, and apelin receptor are underexpressed. Apelin receptor activation has been shown to induce vasodilation by nitric oxide production. This effect has also been observed in diseased vessels, but it is postulated it may be promoted by prostanoids rather than NO. Systemic administering of apelin has been shown to reduce peripheral vascular resistance and blood pressure in both healthy subjects and in those with heart failure, even while the renin–angiotensin system is activated [78,84,85,86].

It is postulated that apelin may play a role in atherosclerosis, but the mechanism has not yet been discovered. In human coronary arteries presenting atherosclerosis, apelin expression is upregulated, and in atherosclerotic plaques both apelin and its receptor can be found (it is not known if this is an antagonistic response to angiotensin II signaling, or due to some pathological mechanisms of plaque formation), but the concentrations of circulating apelin are lower in patients with coronary artery disease, especially in symptomatic cases [78,87].

In myocardial infarction apelin and apelin receptor, both expressed on human platelets, may play a role in reducing platelet aggregation by NO-cyclic guanosine monophosphate signaling. Apelin also inhibits plasminogen activator inhibitor-1 (PAI-1), an inactivator of the endothelium-derived fibrinolytic factor [88,89]. All of the above were presented in animal models; there are no clinical studies available in humans.

The apelin inotropic effect may play a protective role in heart failure conditions. Although more research on this effect is needed, it is suggested that enhanced inotropy may be due to increasing of calcium sensitivity in cardiomyocytes. Activation of extracellular signal-regulated kinase 1/2 (ERK1/2), phospholipase C, and protein kinase C pathways by apelin in turn leads to activation of the myosin light chain kinase and inhibition of troponin I phosphorylation. Both these effects enhance calcium sensitivity in myofilaments. It is important to note that in healthy heart tissue, apelin is not present, but in patients with heart failure, both apelin and the apelin receptor have been found [78,90,91,92].

There is evidence that the concentration of apelin decreases with age, which along with a decreased level of the angiotensin 2 conversion enzyme (ACE2) results in a deepening heart failure [73].

Apelin appears to be a promising potential drug, primarily for treatment of AH and HF. The main obstacle to broader research is the current lack of an oral form of this molecule.

**Figure 5:**
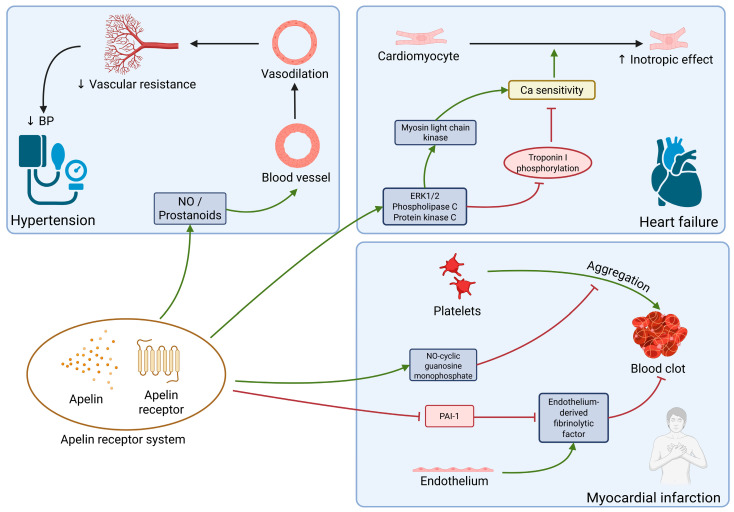
Apelin receptor system plays a role in hypertension, heart failure, and myocardial infarction. In hypertension, acting through nitric oxide or prostanoids, it promotes vasodilation, which in turn lowers vascular resistance and blood pressure. It facilitates its inotropic effect on cardiomyocytes through ERK1/2, phospholipase C, and protein kinase pathways, inducing cardiomyocyte sensitivity to calcium by inhibiting troponin I phosphorylation and activation of myosyne light chain kinase. In myocardial infarction, apelin inhibits PAI-1, and reduces NO-cylic guanosine monophosphate, both of which improve blood flow. ERK1/2: extracellular signal-regulated kinase 1/2; PAI-1: plasminogen activator inhibitor-1; ↑ increase; ↓ decrease.

Apelin’s functions in different diseases is summarized in Figure 5.

### 2.6. FSTL1

Follistatin-like 1 (FSTL1) exhibits a wide expression in many tissues and contributes to the regulation of such processes as the maturation, proliferation, and diversification of various cells [93,94]. FSTL1 is present in high concentration within skeletal muscle cells and plasma following physical exertion, predominantly resistance training [95]. It is the direct antagonist of myostatin, increasing the production rate of muscle cells [96]. A study from 2015 has demonstrated the usefulness of moderate physical effort in preventing and treating cardiovascular diseases [97].

In the case of the heart muscle cells, FSTL1 plays a twofold role: it improves the functioning of the myocardium by stimulating the development and proliferation of cardiomyocytes; however, in certain circumstances it is also a profibrotic factor contributing to myocardial fibrosis [98]. Multiple studies have proved that FSTL1 plays a part in regenerative processes, particularly after an ischemic heart incident: a loss of FSTL1 in such circumstances leads to a slower proliferation of myocytes [99], which is why this particle has been of interest in the context of its therapeutic potential. The promotion of myocyte proliferation by FSTL1 occurs mainly through the activation of Akt, which in turn activates mTOR Complex 2 (mTORC2) signaling [100].

FSTL1 also exhibits antiapoptotic properties: it serves as an activator for the AMP-activated kinase protein (AMPK) and increases its expression [67]. AMPK reacts to damage caused by hypoxia and in low energy conditions is one of the primary elements activating Akt. In ischemic conditions it reduces the area of damage in ischemia-reperfusion injury [101]. Another antiapoptotic factor regulated by FSTL1 is endothelial nitric oxide synthase (eNOS). The activation of eNOS is a significant part of the protective process in hypoxia [102], reducing the effects of oxidation stress and inhibiting apoptosis [103]. FSTL1 indirectly inhibits apoptosis precisely through the activation of the Akt/eNOS/NO trail.

Recent studies have also pointed to FSTL1’s role in the fibrosis of numerous tissues and organs via TGF-beta. The TGF-beta/Smad trail is a classic and well-researched signaling route promoting myocardial fibrosis [104]. This process is known as EMT, epithelial-mesenchymal transition. The activation of heart fibroblasts is triggered by TGF-beta1, and their transformation into myofibroblasts by the activation of Smad 2/3 (classical Smad pathway) [105]. FSTL1 also plays a part in the nonclassical Smad pathway, where the main role belongs to the mitogen-activated protein (MAPK). FSTL1 is activated by TGF-beta1, and in turn, FSTL1 overexpression promotes TGF-beta signaling, which leads to an increased level of MAPK and the stimulation of the signaling path tied to the activation and diversification of fibroblasts via the ERK, JNK, and p38 signals. Removing FSTL1 causes a reduction in ERK, JNK, and p38 levels, but blocking only JNK and p58 also substantially lowers the proliferation, diversification, and migration rate of myofibroblasts [98]. Research has confirmed the participation of FSTL1 in fibrosis by exogenously administering FSTL1, which activates the TGF-beta/Smad trail [98,106], although not all studies corroborate that [107].

It is more and more often postulated that extended, strenuous physical exercise may lead to cardiac dysrhythmia and heart fibrosis [108]. This could occur when FSTL1 activates due to prolonged and taxing physical effort consuming large quantities of energy and stimulates the TGF-beta path [98,109].

**Figure 6:**
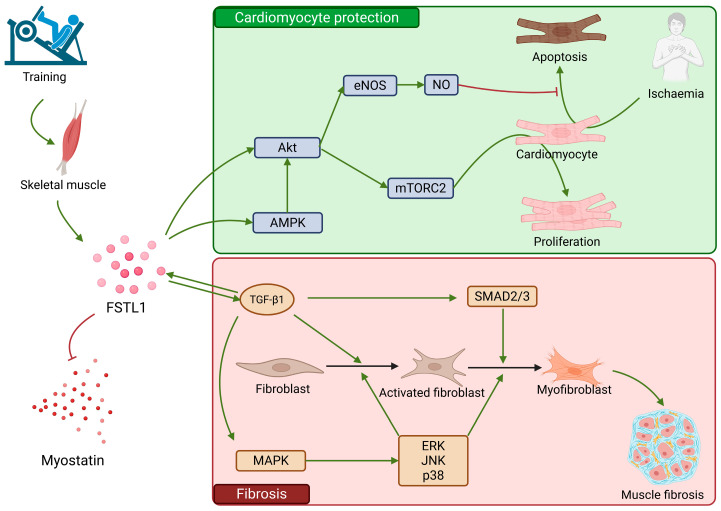
FSTL1, expressed by skeletal muscle during training, can act in two-fold ways: it can protect cardiomyocytes during ischemia, but also can act as a profibrotic factor. Through Akt and AMPK signaling, it prevents muscle apoptosis through the AKT/eNOS/NO pathway, and through mTORC2 it promotes muscle proliferation. By acting through TGF-β1 (which also promotes FSTL1 expression) it activates fibroblasts and promotes their diversification, which leads to muscle fibrosis. It does this by activating the SMAD2/3 pathway, and MAPK, which promotes ERK, JNK, and p38 signaling. FSTL1 is an inhibitor of myostatin. Akt: kinase B protein; AMPK: AMP-activated kinase protein; eNOS: endothelial nitric oxide synthase; mTORC2: mTOR Complex 2; MAPK: mitogen-activated protein; ERK: extracellular signal-regulated kinase; JNK: c-Jun N-terminal kinase.

Both the beneficial and harmful effects of FSTL1 have been summarized in Figure 6.

### 2.7. Myostatin

Myostatin is a myokine of the transforming growth factor-beta (TGF-beta) family, whose main roles are inhibiting the growth of skeletal muscles and reducing insulin resistance [110]. In normal conditions, the expression of myostatin is mostly limited to skeletal muscles and, to a much lesser extent, adipose tissue [1]; however, developing HF increases its expression in those tissues, as well as in the myocardium and circulating blood [111].

By working in opposition to the insulin-like growth factor-I (IGF-I), myostatin protects the myocardium in ischemic conditions and prevents the development of heart failure and muscle hypertrophy [112]. However, overexpression of myostatin in the heart leads to heightened fibrosis processes and the loss of myocytes along the signaling path TAK-1-MKK3/6-p38 [113].

Myopathy and sarcopenia both trigger myostatin overexpression, leading to a strong activation of the Smad2/3 path, which increases proteasomal and autophagic-lysosomal capabilities [111,112,114]. In patients with HFrEF, sarcopenia, as a strong predictor of disability and mortality, occurs as a result of numerous molecular mechanisms, including Smad2/3 signaling [114]. It is possible that these inconsistencies with regards to the protective properties of myostatin pertain to the etiology of HF, both ischemic and otherwise [115].

There are conflicting reports on the level of myostatin in patients with CHF. In the study by Chen et al. [14], patients with a higher concentration of NT-pro BNP and a higher class of NYHA also exhibited a respectively higher concentration of myostatin in circulating blood [14]. The increased concentration of myostatin was related to muscle atrophy and heart failure, and could possibly contribute to the pathogenesis of CHF [14,116]. The heightened activity of myostatin in CHF may be caused by the increased strain on the myocardium and the elongation of myocytes [117]. A publication by Furihata et al. is in opposition to the majority of recently published research on the matter; it reports a decrease in myostatin expression in patients with CHF [118]. This, however, may have to do with the composition of the researched group, comprised of patients with stable CHF, 70% of whom were actively exercising during the examination.

Chen et al. [14] did not find the myostatin level to be dependent on the type of HF (systolic or diastolic). A study by Bączek et al. [119] conducted on a group of geriatric patients demonstrated a significantly higher concentration of myostatin in patients with HFrEF compared to those with HFpEF [119].

Research on animal models indicated myostatin as an aggravating factor in the development of sarcopenia and heart failure. In mice, deletion of myostatin genes in the myocardium protected skeletal muscles from atrophy [120]; it was also observed that the absence of myostatin improved heart function after an infarction, reducing the area afflicted by fibrosis [121].

It is known that chronic heart failure correlates with gradual loss of skeletal muscle mass; therefore, whenever the analysis of myokines activity is studied in these conditions it would be important to assess the examined patients’ muscle mass, their everyday activity, the type of EF (HFpEF or HFrEF), and comorbidities. The number of conflicting reports requires a cautious approach to evaluating the role of myostatin in the development of heart failure of different etiologies and extrapolating the results of research on animal models to the human population.

**Figure 7:**
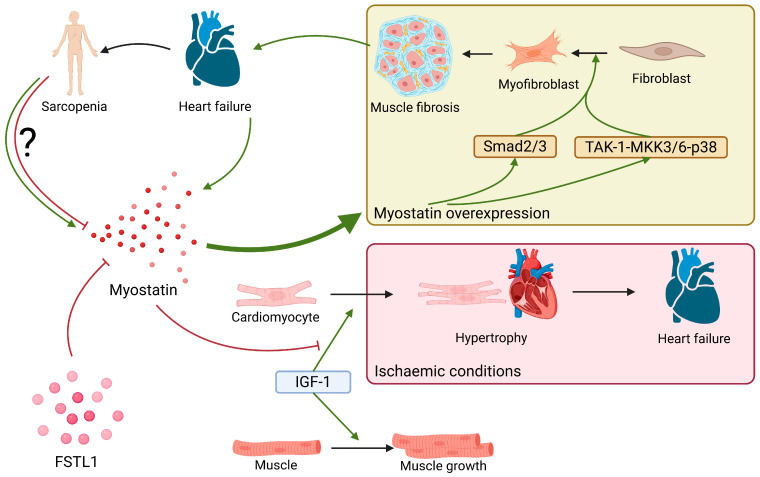
Myostatin expression is seen in patients with heart failure, but there is conflicting data on whether sarcopenia is a promoting or inhibiting factor on myostatin expression. FSTL1 is a known myostatin inhibitor. In ischemic conditions, myostatin may act in opposition to IGF-1 and prevent cardiac muscle hypertrophy and therefore prevent heart failure worsening. However, if overexpressed, acting through Smad2/3 and TAK-1-MKK3/6-p38 pathways it promotes fibroblast activation and differentiation leading to fibrosis, which in turn worsens heart failure; ?: unknown relation.

Summary of myostatin functions described above can be found in Figure 7.

## 3. RA as a Risk Factor of Heart Failure and Sarcopenia

Observations of RA patients spanning multiple years have indicated a positive correlation between that condition, heart failure, and decrease in muscle mass. There are some risk factors common to both sarcopenia and HF. Traditional factors, independent from any coexisting chronic inflammatory disease, include smoking tobacco, low physical activity, and insulin resistance or fully developed type-2 diabetes. However, in the case of RA there are additional factors negatively affecting skeletal muscles and the myocardium. These include chronic inflammation as an independent predictor of cardiovascular incidents [122,123], as well as an altered lipid profile (a lower level of total cholesterol and LDL fraction in RA patients compared to the general populace entails a higher cardiovascular risk [124,125,126]). Moreover, frequent use of GKS or NSAIDs drugs by patients with RA also increases the risk [122].

An analysis of data on the US populace gathered by the National Health and Nutrition Examination Survey in 1990–2020, published in 2024 by Kadier et al. [127], indicated a significant correlation between persons with diagnosed RA and the coexistence of HF. The analysis of data on 37,736 persons demonstrated an almost doubled risk of HF in patients with RA. It is worth noting that this correlation did not apply to patients with diagnosed seronegative RA: here the risk of HF was not increased [127]. Similar conclusions were drawn by Mantel et al. [128] in their analysis of data on the Swedish populace published in 2017: in the cohort of patients with newly developed RA, the total risk factor for subsequent HF of any type was between 1.22 and 1.27, while the risk of non-ischemic HF increased dramatically after the occurrence of RA, which was not the case for ischemic HF. High activity of the disease was observed in all types of HF, but it was the most prominent in the case of non-ischemic HF. In the cohort of patients with ongoing RA, the risk factors for various subtypes of HF were between 1.71 and 1.88. Here, too, the risk of HF was not increased in patients with seronegative RA [128].

Disorders in the structure and function of skeletal muscles in patients with RA differ depending on the applied treatment. Muscle fibers in patients with RA from before the introduction of bDMARDs were described as smaller (especially type II fibers) [129,130], with a lower number of type I fibers compared to type II [131]. Nowadays, in the era of DMARD availability, there are no visible changes in the composition of muscle fibers. The presence of such changes may indicate inadequate treatment and often signifies aggravation of RA [132,133], while sarcopenia appears in the case of high disease activity [134,135,136]. However, impairment of muscular function is more often observed in patients with RA when sarcopenia coincides with excessive adipose tissue [134,137]. Fat gathers as both intra- and intermuscular tissue (the extent of these changes depends on the activity of the disease), causing the muscle tissue composition to resemble that of a person 15 years older [138,139]. In a study conducted on a group of 320 patients with prolonged RA, sarcopenia was diagnosed in 2.2–6.6% cases (depending on the employed criteria), and a loss of muscle mass in 16.9%. A higher risk of sarcopenia correlated with factors such as sex (male), age, and low BMI index [140].

Although there is a link between rheumatoid arthritis, sarcopenia, and heart failure, and the above studies examine these connections, the role of myokines, which appear to contribute to the worsening of each of these conditions, there are no specific studies focusing on this specific link: that is a limitation of this review. We know that myostatin, irisin, and apelin levels are altered in RA. We also know that these myokines contribute to the development of CVDs. Therefore, additional studies on those myokines, as well as the others discussed in this article, in patients with these specific comorbidities could hopefully provide more detailed answers.

## 4. Materials and Methods

To compose this review, we searched through PubMed and Google Scholar databases for English publications containing the following keywords: “cardiovascular diseases”, “heart failure”, “myocardial infarction”, “coronary heart disease”, “hypertension” “myokines”, “rheumatoid arthritis and cardiovascular diseases”, “rheumatoid arthritis and sarcopenia”, “myokines and rheumatoid arthritis”, “dermcidin”, “irisin”, “musclin”, “myonectin”, “apelin”, “FSTL1”, and “myostatin”. Among the studies there are original research papers, editorials, reviews, and meta-analyses.

## 5. Conclusions

Altered concentrations of particular myokines in circulation have been observed at different stages of HF, resulting in abnormal reconstruction of the heart, diastolic dysfunction, lower systolic function, and progressing myopathy of skeletal muscles. Myokines are not only involved in the pathogenesis of skeletal muscle myopathy, but can also provide new information on the development of chronic HF and help identify patients with higher risk of complications.

Persons with diagnosed RA constitute a particular group of patients, in which a disrupted profile of certain myokines coincides with a higher risk of cardiovascular incidents and a pathological remodeling of skeletal muscles. Each new study lets us paint a progressively more detailed picture of the pathophysiological mechanisms behind the coexistence RA and HF; however, there is no research to date examining the role of myokines in cases of such double diagnosis.

Large-scale clinical research could provide a clearer view on the role of myokines in the development of the individual disease entities and their predictive role in HF, as well as distinguish potential therapeutic goals for new drugs, which in turn would have an impact on everyday clinical practice.

## Figures and Tables

**Table 1:** Summary of selected myokines, their point of origin, and involvement in cardiovascular diseases.

Myokine	Organ of Origin	Myocardial Injury	Heart Failure	Arterial Hypertension
Dermcidin	Sweat glands, skeletal muscle	Increased area of ischemia (induction of apoptosis of injured muscle cells)	-	-
Irisin	Skeletal muscle, adipose tissue, myocardium	Protection from apoptosis in early stages, induction of apoptosis in later stages	Downregulated in HFrEF, which may lead to cardiac hypertrophy	Lowering of blood pressure
Musclin	Skeletal muscle, osteoblasts, arteries	-	Protection from fibrosis. Improvement of cardiomyocyte function	Overexpression leads to induction of hypertension
Myonectin	Skeletal muscle, adipose tissue	Inhibition of cardiomyocyte apoptosis. Improvement of endothelial function	Downregulated in developing HF	-
Apelin	Brain, stomach, adipose tissue	Induction of antiplatelet effects. Improvement of endothelial function	Prevention of myocardium hypertrophy. Inotropic effect. Downregulation in HF leads to fibrosis	Antihypertensive mechanisms: diuresis induction, dilation of blood vessels
FSTL1	Numerous tissues, including myocardium and endothelium	Protection from cardiomyocyte apoptosis, induction of myocyte proliferation	Involvement in profibrotic mechanisms	-
Myostatin	Myocardium, skeletal muscle, adipose tissue	Prevention of HF development in ischaemic conditions	Upregulated in HF, induces myocardial fibrosis	-

## Data Availability

No new data were created or analyzed in this study.
